# Elevated relative humidity significantly decreases cannabinoid concentrations while delaying flowering development in *Cannabis sativa* L.

**DOI:** 10.3389/fpls.2025.1678142

**Published:** 2025-11-17

**Authors:** Ingrid Carolina Corredor-Perilla, Tae-Hyung Kwon, Sang-Hyuck Park

**Affiliations:** 1 Institute of Cannabis Research, Colorado State University-Pueblo, Pueblo, CO, United States; 2 Institute of Biological Resources, Chuncheon Bioindustry Foundation, Chuncheon, Republic of Korea

**Keywords:** canopy-range relative humidity, cannabis, vapor pressure deficit (VPD), biomass, morphology, cannabinoids, cannabidiol (CBD), cannabidiolic acid (CBD-A)

## Abstract

**Introduction:**

Relative humidity (RH) is critical for regulating transpiration, plant morphology, and the biosynthesis of secondary metabolites in crops. However, its specific impacts on cannabis (*Cannabis sativa* L.) genotypes, especially concerning optimal growth and cannabinoid concentration, remain inadequately understood.This study aimed to investigate the effects of canopy-level RH on plant development and cannabinoid concentration in a CBD–dominant strain.

**Methods:**

Plants were cultivated under controlled conditions at two distinct RH ranges: low RH (37-58%) and high RH (78-98%). Growth metrics, including stem length, trunk diameter, number of nodes, apical internode spacing, and flowering time, were recorded weekly. Upon floral maturation and harvest, biomass and cannabinoid concentrations were measured. A total of 14 cannabinoids were quantified via high-performance liquid chromatography (HPLC) to assess compositional shifts under different RH conditions.

**Results:**

Cultivation under high RH resulted in a reduced vapor pressure deficit (VPD) ranging from 0.62 kPa to 0.25 kPa during flowering, indicating values outside the optimal range for cannabis cultivation. This environment led to significant reductions in total biomass (-75.3%), flower biomass (-71.0%), trunk diameter (-0.4%), and node count (29.3%), compared to low RH conditions (n = 10 per range, *p* < 0.001). Conversely, stem length increased by 9.7%, and apical internodal spacing expanded by 0.04% under high RH (n = 10, *p* < 0.0001). Flowering was delayed by three weeks with high RH, accompanied by notable reductions in both vegetative growth and inflorescence production. Furthermore, high RH significantly suppressed cannabinoid accumulation: cannabidiolic acid (CBD-A), cannabidiol (CBD), and cannabichromenic acid (CBC-A) levels decreased by approximately 4.9-fold, 3.2-fold, and 13-fold, respectively. Total cannabinoid concentrations of CBD and CBC were similarly diminished by 4.6-fold and 1.5-fold (n = 10, *p* < 0.0001).

**Discussion:**

This study highlights that elevated canopy-level humidity, outside optimal VPD thresholds, can significantly delay flowering, reduce biomass accumulation, and negatively impact cannabinoid concentrations in *Cannabis sativa* L. cv. Cherry Berry.

## 1 Introduction

*Cannabis sativa* L. is an annual and dioecious flowering plant traditionally cultivated for fibers, seeds, oil production, and medicinal purposes (Innes and Vergara, 2023). The plant’s adaptive traits have been crucial in sustaining human societies for thousands of years. However, recent accelerated domestication, primarily targeting increased fiber and cannabinoids yields, has reduced genetic variability and divergence while enhancing yield potential (Ren et al., 2021).

The growth and development of *Cannabis sativa* are primarily influenced by genetic factors; however, environmental conditions and management practices also play a critical role. Numerous studies have provided valuable insights into the impact of environmental variables, including light intensity, wavelength, photoperiod, temperature, drought, rainfall, flooding, and relative humidity, on cannabis growth and cannabinoid concentration (Alter et al., 2024; Carranza-Ramírez et al., 2025; Fleming et al., 2023; Holweg et al., 2025; Park et al., 2022; Rodriguez-Morrison et al., 2021; Preprint Shenhar et al., 2025). While light and photoperiod are discussed in detail below to provide necessary context, this study focuses on relative humidity, which remains a comparatively underexplored environmental factor in cannabis cultivation. Among these factors, relative humidity remains one of the least studied despite its significant effects on plant growth and cannabinoid biosynthesis.

Light intensity affects cannabis genotypes differently, with distinct responses observed among chemotypes. For example, Δ^9^-tetrahydrocannabinol (THC)-dominant chemotype exhibits increased leaf-level of photosynthesis, and improved water-use efficiency, when Photosynthetic Photon Flux Density (PPFD) levels were increased between 1,600–2,000 µmol m^−2^ s^−1^ (Chandra et al., 2015). However, these measurements do not necessarily reflect the final yield and crop performance. Similarly, ‘Critical CBD’, characterized by a THC/CBD (cannabidiol) ratio of 0.5, exhibited abundances exceeding 41% for CBD, THC, cannabinol (CBN), cannabichromene (CBC), cannabigerol (CBG), and tetrahydrocannabivarin (THCV) under a PPFD of 1,000 µmol m^-^² s^-^¹ compared to 600 µmol m^−2^ s^−1^ (Sae-Tang et al., 2024). In the high-THC genotype ‘Meridian’, the same PPFD increased cannabinoid yield in biomass 1.5 times, dry weight 1.6 times, and harvest index by 7% compared to 600 µmol m^−2^ s^−1^ (Llewellyn et al., 2022). Notably, THC-rich genotype ‘Gelato’ showed well-saturated leaf photosynthesis and linear increase in dry inflorescence weight as PPFD increased from 120 to 1,800 µmol m^−2^ s^−1^. No adverse effects on cannabinoid potency were observed, with inflorescence yields reaching 501 g m^-2^, total cannabinoid concentration of 83.5 g m^-2,^and a total equivalent cannabidiolic acid (CBD-A) content of 214 mg g^-1^ of dry inflorescence at the highest PPFD level (Rodriguez-Morrison et al., 2021).

As light intensity influences cannabinoid concentration, the light spectrum also plays a critical role in cannabis cultivation. Studies investigating blue:red light ratios (1:1 and 1:4) in comparison to full-spectrum LED lighting revealed higher inflorescence yield, alongside notable alterations in plant morphology, physiology, biomass accumulation, and cannabinoid composition, including cannabigerolic acid (CBG-A), CBD, THC, and CBC (Danziger and Bernstein, 2021). These effects varied by genotype, particularly among genotypes with differing TCH-A:CBD-A ratios, underscoring the importance of genetic background in plant responses (Danziger and Bernstein, 2021). Studies investigating the red to far-red (R:FR) ratio (1:11) in CBD-rich cannabis genotypes significantly increased the concentration of CBD (0.035%), CBG-A (0.017%), and THCV-A (0.033%) compared to a low R:FR ratio (1:1) (Kotiranta et al., 2024). In contrast, UVA and UVB exposure have demonstrated no significant effects on biomass accumulation or cannabinoid concentration in indoor-grown THC-dominant genotypes (Llewellyn et al., 2022). Additionally, LED lighting with a low blue-to-red ratio has been shown to influence plant morphology, improved photosynthetic efficiency, and modulate both CBD and THC content in non-psychoactive hemp varieties (Carranza-Ramírez et al., 2025).

Beyond light intensity and spectral quality, photoperiod critically influences cannabis growth, physiology, and cannabinoid biosynthesis (Ahsan et al., 2024). As a facultative short-day genotype- species, cannabis requires day lengths between 9 and 15 hours to initiate flowering (Dowling et al., 2021, 2024). This latter process is governed by internal regulatory mechanisms such as gibberellic acid (GA) signaling, circadian rhythm, flowering-related gene expression, and modulated by external cues including nutrient availability, plant architecture, and ambient temperature (Ahsan et al., 2024; Alter et al., 2024; Spitzer-Rimon et al., 2019, 2022). Recent studies on photoperiod in cannabis genotypes have shown that a minimum of three days of short photoperiod exposure, accompanied by reduced levels of GA_4_ and auxin at the shoot apex, is required to initiate and sustain inflorescence formation (Alter et al., 2024).

Interestingly, the initiation of solitary flowers and bract development can still occur under long-day photoperiods, suggesting partial independence from photoperiodic cues (Alter et al., 2024; Spitzer-Rimon et al., 2022).

A recent study has shown that a 12-hour photoperiod effectively optimizes CBD production, increasing its concentration while simultaneously enhancing dry biomass yield (Xu et al., 2024). Similarly, tropical hemp genotypes exhibited elevated levels of both CBD and THC under a 12.5-hour photoperiod, whereas temperate genotypes showed minimal sensitivity to changes in photoperiod (De Prato et al., 2022).

In fiber-type cannabis varieties, the combined effects of photoperiod and light spectrum were assessed using white LED light (380–750 nm) and purple LED light (200–400 nm). A 16-hour photoperiod of white light followed by eight hours of darkness increased photosynthetic efficiency, while purple light improved photoprotective responses (Šrajer Gajdošik et al., 2022). In contrast, 24-hour light exposure, regardless of the light type, caused thylakoid membrane damage, underscoring the need for further research on antioxidant responses and light-induced stress (Šrajer Gajdošik et al., 2022). Synergistic effects of photoperiod and light regimes play significant roles in the physiology of cannabis varieties for industrial uses, altering plant development.

Temperature greatly affects cannabis cultivation, with optimal growth at 25–35 °C. In the low-THC hemp genotype ‘V4’, a moderate day/night temperature regime (day: 27 °C/night: 21 °C) resulted in the highest concentrations of cannabinoids in dry-weight inflorescences. Under these conditions, the levels of CBD-A (40.3 mg g−1), CBC-A (2.60 mg g−1), and THC-A (2.12 mg g−1) increased by 28.75%, 43.6% and 41.3%, respectively, compared to the values of a constant 24 °C day/night schedule (Bok et al., 2023). In contrast, for THC- and CBD-rich genotypes, increasing day/night temperatures from 25 °C/21 °C to 31 °C/27 °C under a short-day photoperiod reduced total cannabinoid yields from over 400 and 200 g m^-2^ to less than 100 g m^-2^, at a PPFD of 1,200 µmol m^−2^ s^−1^ (Holweg et al., 2025). The study concluded that elevated temperatures also disrupted inflorescence development, decreased biomass, and altered cannabinoid concentration (Holweg et al., 2025).

Heat stress is a common environmental challenge in cannabis cultivation, yet its effects on cannabinoid concentration and gene expression remain relatively underexplored. In a study of 75-day-old plant leaves of industrial genotypes, ‘Hot Blonde’, ‘Cherry Blossom’, and ‘Queen Dream’, exposure to heat stress of 45 °C-50 °C for 48 hours resulted in increases in CBD levels by 38.4-fold, 35.07-fold, and 22.92-fold, respectively, and in CBN by 5.08-, 13.40-, and 11.05-fold, respectively (Hahm et al., 2025). These temperature ranges also increased the expression of *MYB, AP2, OLS, OAC, PT, THCAS, CBDS*, and *CBCAS* in the terminal inflorescences (Hahm et al., 2025). Similarly, Park et al. (2022) reported that CBD-rich genotypes exposed to sustained heat (45–50 °C) for seven days exhibited an 83% decrease in CBG-A and a 40% increase in CBG in immature hemp flowers (Park et al., 2022).

Cold stress adversely affects secondary metabolism, physiology, and yield in cannabis. In hemp genotypes, exposure to 4 °C for 7–14 days led to cell membrane damage, as indicated by electrolyte leakage. In contrast, tolerant genotypes exhibited upregulation of cold-response (*COR*) genes and activation of DNA methylation pathways (Mayer et al., 2015). Likewise, in CBD-dominant genotypes exposed to different combinations of cold stresses. Some plants were acclimated to cooler temperatures of 10 °C, then all plants were exposed to freezing stress 0 to 3 times at -0.5% for three hours per event. Acclimated and non-acclimated plants reduced cannabinoid concentrations of CBD and THC, with limited frost tolerance (Galic et al., 2022). In contrast, cold stress at 4 °C for 0, 12, 24, and 48 hours did not produce significant changes in their cannabinoid concentration in industrial genotypes (Hahm et al., 2025). In addition, moderately cool temperatures (8–15 °C) improved anthocyanin accumulation, though CBD yield and biomass depended more on maturity (Kim, 2024). Under 4 °C, lipidomic analysis revealed dry weight reductions (37% to 22%), increased osmoprotectants and stress enzymes, and membrane remodeling with galactolipids reaching 70% of total lipids (Yan et al., 2025). Collectively, these findings illustrate the complex molecular and physiological responses and strategies of *Cannabis sativa* genotypes to low-temperature stress.

In addition to thermal stress, water stress substantially alters cannabinoid accumulation and plant biomass in *Cannabis sativa*. Under moderate drought conditions, defined by a 50% reduction in transpiration compared to well-watered controls, CBD and CBG-A levels increased by approximately 25% and 10–15%, respectively (Dimopoulos et al., 2024). In substrates with low water retention, the production of the PIP (Intrinsic plasma membrane proteins) subfamily was augmented, particularly the aquaporin isoforms PIP1.4, PIP2.3, and PIP2.1, thereby increasing water transport, resulting in greater inflorescence development as well as a 2.8% rise in CBD content (Ortiz Delvasto et al., 2023). In contrast, severe drought stress, marked by a 70-80% reduction in normal transpiration or the complete withholding of water irrigation, caused significant reductions in both cannabinoid concentration and plant biomass. THC-A and CBG-A levels were reduced by 40% and 48% (Preprint Shenhar et al., 2025). Similarly, water stress applied during early flowering led to a reduction of 70–80% in both CBD and THC content (Park et al., 2022). Despite these adverse effects, certain genotypes such as Ivory and Santhica 27 demonstrated resilience by maintaining relatively high biomass yields under dry conditions (Herppich et al., 2020).

Although a wide range of environmental factors influencing cannabis cultivation has been well documented, the role of relative humidity (RH) remains comparatively understudied. RH is defined as the ratio of atmospheric vapor pressure to saturation vapor pressure at a given temperature (Campbell and Norman, 1998; Fairbridge, 1987). This parameter reflects the degree of air saturation with water vapor, and has a direct effect on key physiological processes, including transpiration rates, stomatal behavior, and vapor pressure deficit (VPD) level, which collectively regulate plant water status and photosynthetic performance (Taiz et al., 2015).

Beyond its role in water relations, RH also plays a crucial role in determining plant morphology, biomass accumulation, nutrient uptake, pathogen development, and secondary metabolite production (Grossiord et al., 2020; Gui et al., 2021; Mortensen, 1986). Optimal RH requirements for *C. sativa* vary by developmental stage, with recommended ranges of 65-75% during the clonal and seedling stages, 50-70% during vegetative growth, and 40-60% during the flowering period (Chandra et al., 2017; Fleming et al., 2023; Jin et al., 2019).

While direct studies on *C. sativa* are limited, research in other plant species suggests that RH can significantly influence physiological and biochemical traits. For example, high RH levels of approximately 80% have been shown to increase leaf biomass and modify nutrient content in tomato plants (Suzuki et al., 2015). Similarly, a study on the effects of climate change, involving night-warm temperatures and elevated atmospheric water saturation, negatively impacted the flowering responses in 184 plant species from the Amazonian forest, decreasing flowering biomass (Vleminckx et al., 2024). Likewise, elevated humidity in silver birch (*Betula pendula*) has been associated with changes in hydraulic architecture, leaf morphology, and metabolite profiles, ultimately contributing to improved stress resilience (Lihavainen et al., 2016; Sellin et al., 2015). Interestingly, studies on high RH of 80% at a constant temperature in petunia showed longer vegetative stages and delayed flowering development (Hoang and Kim, 2018). These findings suggest that *C. sativa* may exhibit comparable responses to RH variation, potentially affecting both biomass production and cannabinoid biosynthesis.

In the context of ongoing climate change, increasing global temperatures and altered precipitation patterns are leading to elevated atmospheric humidity levels (Lahlali et al., 2024). In regions such as Colorado, where *Cannabis* cultivation is widespread, these environmental changes highlight the importance of identifying and cultivating genotypes that can perform well under variable RH conditions ((EPA) United States Environmental Protection Agency, 2016). Despite this need, the direct effects of RH on *C. sativa* genotypes adapted to temperate climates have not been thoroughly investigated.

Therefore, this study aims to assess the impact of canopy-relative humidity on the morphometry, biomass production, and cannabinoid concentration of a type III CBD-dominant chemotype under controlled conditions. The findings will contribute to optimizing cultivation practices and enhancing quality and yield in commercial hemp production.

## 2 Materials and methods

### 2.1 Plant material and cultivation

A type III CBD-dominant chemotype that typically produces between 14–18% CBD and contains 0.2–0.3% THC was used. Uniform clones (10–12 cm in length) were taken from a six-month-old mother plant. Cuttings were treated by dipping the basal end in a 0.3% indole-3-butyric acid (IBA) gel (Clonex^®^) (Growth Technology Ltd., Vista, CA), and subsequently inserted into (stone wool) rock wool cubes (Root Riot^®^) (Hydrodynamics International, Medford, OR) within an aeroponic cloning system following the procedure and recommendations of Regas et al., 2021) (**Supplementary Table 1**). A nutrient solution composed of a three-part formulation (General Hydroponics^®^: FloralGro^®^, FloralMicro^®^, FloralBloom^®^) was manually applied over a four-week rooting period following the commercial recommendation of dosage for every stage, from clones, vegetative, and flowering stages (**Supplementary Table 1**).

After the root system reached 25–30 cm in length, the plantlets were transplanted into 3.78 l soil pots for two-week acclimation. A mixture of soils and substrates was used during the plant’s growth (**Supplementary Table 1**); each plant received 900 ml of nutrient solution twice to three times a week during the plant’s growth (**Supplementary Table 1**). Plants were grown without trimming or insecticide spray to prevent any unintended effects on growth and secondary metabolism.

The study used a randomized complete block design with 20 feminized type III CBD-dominant clones, grown under two canopy-level relative humidity (RH) conditions: low RH (37–58%) and high RH (78–98%). Each treatment group included ten plants placed in growth chambers (SciBrite^®^, Percival, LED36L1-120V; Percival, Iowa, U.S.) with an interior volume of 0.84 m³ and outside dimensions of 85.09 cm (width) × 85.34 cm (length) × 196.09 cm (height).

Since these chambers lack integrated humidity control systems, RH levels were adjusted manually. To achieve low RH, the chamber door was left slightly open, and the lid was adjusted to create a small 0.5 cm opening, allowing more air exchange with the outside and preventing moisture buildup inside. For high RH, the chamber was fully sealed, and an additional 900 ml container of water was placed near the soil pots to increase moisture around the plant canopy.

The study followed a randomized complete block design using 20 feminized type III CBD-dominant clones, grown under two canopy-level relative humidity (RH) treatments: low RH (37–58%) and high RH (78–98%). Each treatment group consisted of ten plants housed in growth chambers (SciBrite^®^, Percival, LED36L2-120V; Percival, Iowa, U.S.) with an interior volume of 0.84 m³ and exterior dimensions of 85.09 cm (width) × 85.34 cm (length) × 196.09 cm (height).

Because these chambers do not have integrated humidity control systems, RH levels were manually manipulated. The low RH condition was achieved by leaving the chamber door slightly open and adjusting the lid to create a controlled 0.5 cm opening, which allowed for greater air exchange with the external environment and prevented moisture accumulation inside the chamber. Conversely, for the high RH condition, the chamber was fully sealed and additional 900 ml containers of water were placed near the soil pots to increase moisture around the plant canopy.

The low-humidity range was selected as the control treatment, reflecting the typical growing conditions used by Colorado growers for this genotype. During the experiment, the growth chambers were set to control light, temperature, and photoperiod. Each chamber was equipped with a 30.48 cm AeroWave A6 fan (Vivosun, Ontario, California, U.S.), operating at 60 Hz to maintain consistent air circulation, with one fan in each chamber.

Morphometric and biomass variables, including stem height, node count, trunk diameter, internodal spacing, were measured weekly and biomass weights (wet and dry) were measured at the end of the 14 (low RH) and 15 (high RH) weeks. The total above- and below-ground dry biomass and biomass by structure were recorded independently for each plant (e.g., inflorescences, leaves, branches, roots, and stems). Cannabinoid concentrations of 14 cannabinoids, expressed as a percentage (% W/W) and mg g ^-1^ of each cannabinoid based on dry weight in the harvested inflorescences, were measured using High-Performance Liquid Chromatography (HPLC). The cannabinoids, including acidic, decarboxylated, oxidized, and degraded forms of cannabigerol (CBG), cannabidiol (CBD), tetrahydrocannabinol (THC), cannabichromene (CBC), tetrahydrocannabivarin (THCV), cannabidivarin (CBDV), the oxidized form of Δ^9^-THC (Δ^8^-THC), and degraded forms such as cannabinol (CBN) were studied. Analyses were performed using HPLC with three replicates per plant (n=90), totaling 180 analysis replicates (Global Hemp Innovation Center, Oregon State University, 2022).

During the 8-week vegetative stage, plants received General Hydroponics Floral Series^®^ as a soil drench (900 ml per plant, three times weekly), with solution volumes adjusted for growth stages. During the 6-week flowering stage, the same nutrient package was applied 2–3 times weekly (900 ml per plant) until 40–70% of the inflorescence developed an amber color for harvesting (Punja et al., 2023; Tran et al., 2025).

Harvesting and Post-Harvesting: Above-ground and below-ground biomass were collected in weeks 14 (low RH) and 15 (high RH), and both wet and dry biomass were recorded. Plants were dried upside down in a dark room (T: 15–21 °C, RH: 40–55%) for 15 days, until brittle (~11%) (Das et al., 2022). Storage moisture was maintained at approximately 80% (Das et al., 2022; Lazarjani et al., 2021). Whole trimmed inflorescences per plant were wrapped in kraft paper and plastic bags and stored in the dark at room temperature (15-18 °C) until cannabinoid analysis. Plant root biomass maintained in trays was gathered using 3- and 12-mm sieves (Gilson, Lewis Center, OH) and rinsed thoroughly until soil and substrate residue were removed. Roots were exposed to sunlight and ambient temperature until they became brittle, a process that took 15–20 days.

#### 2.1.1 Photoperiod and light regimen

PPFD was monitored throughout all growth stages using a PAR Meter (Photobio Quantum) (Phantom, Hydropharm, Shoemakerville, PA). During rooting in aeroponics, plants were exposed to a 24-hour light cycle with AgroBrite T5 lights at 200-225 μmol m^-^² s^-1^for four weeks. During the acclimation stage, a 24-hour photoperiod with a PPFD of 300 μmol m^-^² s^-1^was maintained for two weeks. For the early vegetative stage, a 15-hour light/9-hour dark cycle was applied for four weeks, with a PPFD of 400-600 μmol m^-^² s^-1^. In the late vegetative stage, the photoperiod was shifted to 13.5 hours light/10.5 hours dark for another four weeks, maintaining the same PPFD. During the early flowering stage, a 12.5-hour light/11.5-hour dark cycle was implemented for three weeks with a PPFD of 400-600 μmol m^-^² s^-1^, corresponding to pistil formation. Finally, at the flowering stage, a PPFD of 600–800 μmol m−2 s−1 was applied for three weeks, ending when approximately 70% of the inflorescences had turned amber.

### 2.2 Environmental parameters in controlled conditions

Environmental parameters, including temperature (T), dew point (DP), humidity ranges, soil temperature, and conductivity, were continuously (hourly) monitored throughout the plant growth stages (**Supplementary Table 2**). Hourly canopy-level data were collected using a data logger (EL-USB-2-LCD) (Lascar, Erie, PA). Soil conductivity and temperature were measured weekly with the Gro Line Soil Test™ Direct Soil Conductivity Tester (HI98331) (Hanna Instruments, Woonsocket, RI). Two canopy relative humidity ranges were tracked hourly through the entire study (14–15 weeks), with ranges of low RH (37-58%) and high RH (78%-98%).

The Vapor Pressure Deficit (VPD) and Leaf Vapor Pressure Deficit (LVPD) were initially estimated by calculating the Saturated Vapor Pressure (SVP), which is the pressure exerted by water vapor in the air when it is saturated at 100% RH at a specific T (Equation 1). The VPD is determined based on temperature and canopy relative humidity data, which identifies the quantity of water vapor in the air at a given RH (Equation 2) (Grossiord et al., 2020). The LVPD is defined as the difference between the SVP inside the leaf and the actual vapor pressure of the surrounding air (Equation 3). Psychrometric tables specific to cannabis, based on parameters defined by Breit et al. (2019), were referenced to determine optimal growth conditions. The VPD and LVPD were calculated using the formulas provided by Breit et al. (2019) and Grossiord et al. (2020):

(1) $$\text{SVP}=610.78\times {\text{e }}^{\frac{\text{T}}{\left(\text{T}+238.3\right)}\times 17.2694}$$

Where SVP represents the saturated vapor pressure, e= 2.71828 is a Mathematical constant of Euler’s Number (e), T is the temperature in °C, the SVP result should be given in pascals, then divided by 1000 to get kPa.

(2) $$VPD=\frac{100-RH}{100}\times SVP$$

Where VPD represents the vapor pressure deficit, RH is the relative humidity, and SVP represents saturated vapor pressure.

(3) $$\text{LEAF VPD}=\text{LSVP}-\frac{\left(\text{ASVP}\times \text{RH}\right)}{100}$$

Where Leaf VPD represents the leaf vapor pressure deficit, ASVP represents the adjusted saturated vapor pressure, and ASVP represents the assumption that leaf and ambient temperature difference is between 1-3°C.

### 2.3 Cannabinoid analysis

#### 2.3.1 Sample preparation

To determine the cannabinoid concentration of the selected genotype, inflorescences from both high RH and low RH at the canopy level were analyzed using liquid chromatography on a Thermo Scientific Dionex UltiMate 3000 HPLC system (Thermo Fisher Scientific, Waltham, MA). For sample preparation, dried flowers were ground with a mortar and pestle and then sieved through a wire mesh with a pore size of 1.18 mm to remove plant debris. The extraction process followed the methods outlined by the Standard Operating Procedures for Hemp – Cannabinoid Analyses (Global Hemp Innovation Center, Oregon State University, 2022).

In each case, 500 mg of genotype samples from both canopy RH ranges were added to a 5 ml glass scintillation tube containing 5 ml of methanol: chloroform (9:1 v/v). Samples were vortexed for 10 seconds, followed by 5-minute sonication in a bath at 40 kHz (Branson 3510) (Branson Ultrasonics Corp, Brookfield, CT) for 5, 10, and 15 minutes. Subsequently, a final centrifugation at 1900 × g for 15 minutes was conducted (Ultra-8V) (L-W Scientific, Lawrenceville, GA) (UNODC, United Nations of Drugs and Crime, 2009). The samples were left overnight for 18 hours in the darkness at room temperature. The supernatant was then collected and filtered using HPLC natural hydrophilic × Nylon 66 membrane syringe filters with a diameter of 13 mm and a pore size of 0.45 μm for sterilization and removal of plant debris (Global Hemp Innovation Center, Oregon State University, 2022).

Three replicates were prepared per plant (n = 10 plants per canopy relative humidity condition, total = 90 samples). For the final solution preparation, each replicate underwent two dilutions of 1:10 (900 μl of methanol and 100 μl of the sample) and 1:100 (990 μl of methanol and 10 μl of the sample) (UNODC, United Nations of Drugs and Crime, 2009).

#### 2.3.2 High-performance liquid chromatography

This HPLC system operated with a temperature-controlled autosampler, a column oven compartment, and a diode array detector (DAD) set to detect at 220 nm (DAD 3000 and multi-wavelength detector ‘MWD 3000’). It was controlled by Chromeleon 7.2 software, version 7.2 SR5 (Thermo Fisher Scientific, Waltham, MA). The decarboxylation process was not used, and the elution order of CBD, CBN, THC, and THC-A was followed.

The HPLC system included a polar Encapped column with an I.D. of 100 mm x 2.1 mm and a particle size of 2.6 μm, maintained at 50 °C (Accucore AQ C18, Thermo Scientific). A 20 μl sample was injected, and the flow rate through an IntertSustain C18, 3 μm, 2.1 x 100 mm column (GL Sciences, Inc., IntertSustain-ods-3-micron-100-x-2–1 mm) was 1.2 mg l^-1^. Two mobile phases were used: mobile phase A, consisting of 0.1% formic acid in 5.0 mM ammonium formate and ultra-pure water (18 MΩ), and mobile phase B, containing 0.1% formic acid in methanol. Equilibration was reached at 2.5 minutes at 60% B, followed by the gradient: 0–2 min, 70% B; 2–8 min, 75% B; 8–9 min, 100% B; 9–10 min, 100% B; and 10–15 min, 60% B, with a step at 10.5–11 min at 60% B followed by 2.5 min of equilibration at 60% B between injections. The total analysis time was 13.5 minutes. The autosampler chamber and column were maintained at 8 °C and 50 °C, respectively. Under these conditions, CBD-A, CBG-A, CBG, CBD, Δ^9^-THC, and THCA-A eluted at 3.171 min (± 0.050 min), 3.401 min (± 0.050 min), 3.611 min (± 0.050 min), 3.673 min (± 0.050 min), 5.234 min (± 0.050 min), and 5.860 min (± 0.050 min), respectively. Five-point calibration curves (1, 5, 10, 50, and 100 mg l^-1^) were generated for the cannabinoids at 228 nm using DAD, with peak integrations analyzed using software (Global Hemp Innovation Center, Oregon State University, 2022).

A set of 14 commercial cannabinoids standards solutions GBG-A (99.3%), CBG (99.2%), CBD-A(99.3%), CBD (99.2%), CBC-A (99.8%), CBC (98.8%), THC-A (99.1%), Δ^9^-THC (99.3%), CBDV-A (99.3%), CBDV (99.2%), THCV-A (97.9%), THCV (99.1%) (Cerilliant, San Antonio, TX) were prepared with a volume of 1.0 mg l^-1^ in methanol solution (Cayman Chemical Company, Ann Arbor, MI)genotype. The sample concentration of each cannabinoid is determined from a calibrated linear response curve (R² > 0.99) in the total extraction volume and adjusted according to the sample weight (Global Hemp Innovation Center, Oregon State University, 2022).

Posterior calculations were employed to determine the mg l^-1^, the density, the dilution factor, the mg/hemp, and the total cannabinoid percentage.

Then, we determined the total cannabinoid percentage of CBG, THC, CBD, CBC, THCV, and CBDV in the assessed samples by using the following Equation 4:

(4) $$TC(\%)=\%\frac{w}{w}[N\;C]+(\%\frac{w}{w}[A\;C]x\;\frac{w}{w}[(M\;R\frac{NC}{AC}\;)])$$

Where TC represents total cannabinoid %, NC represents neutral cannabinoids such as CBG, THC, CBD, CBC, THCV, and CBDV, AC represents acidic cannabinoids such as CBG-A, THC-A, CBD-A, CBC-A, THCV-A, CBDV-A, M R = mass ratio, it is the ratio of the molecular weight of cannabinoids; Decarboxylation: acidic cannabinoids that lost a CO_2_ molecule to form neutral structures.

### 2.4 Statistical analysis

Environmental parameters (temperature, humidity, and dew point) were monitored hourly and averaged for the vegetative stage (8 weeks) and total growth period (14–15 weeks). Morphometry, biomass, and cannabinoid data were analyzed using unpaired t-tests to detect significant differences between RH at canopy levels. One-way ANOVA evaluated individual cannabinoid differences (% w/w) across humidity conditions. Data analysis and visualizations were conducted in GraphPad Prism 9^®^ (GraphPad Software, Inc., La Jolla, Ca).

## 3 Results

### 3.1 Canopy humidity-induced variations in temperature, dew point, and vapor pressure deficit during *cannabis* growth

**Figure 1:**
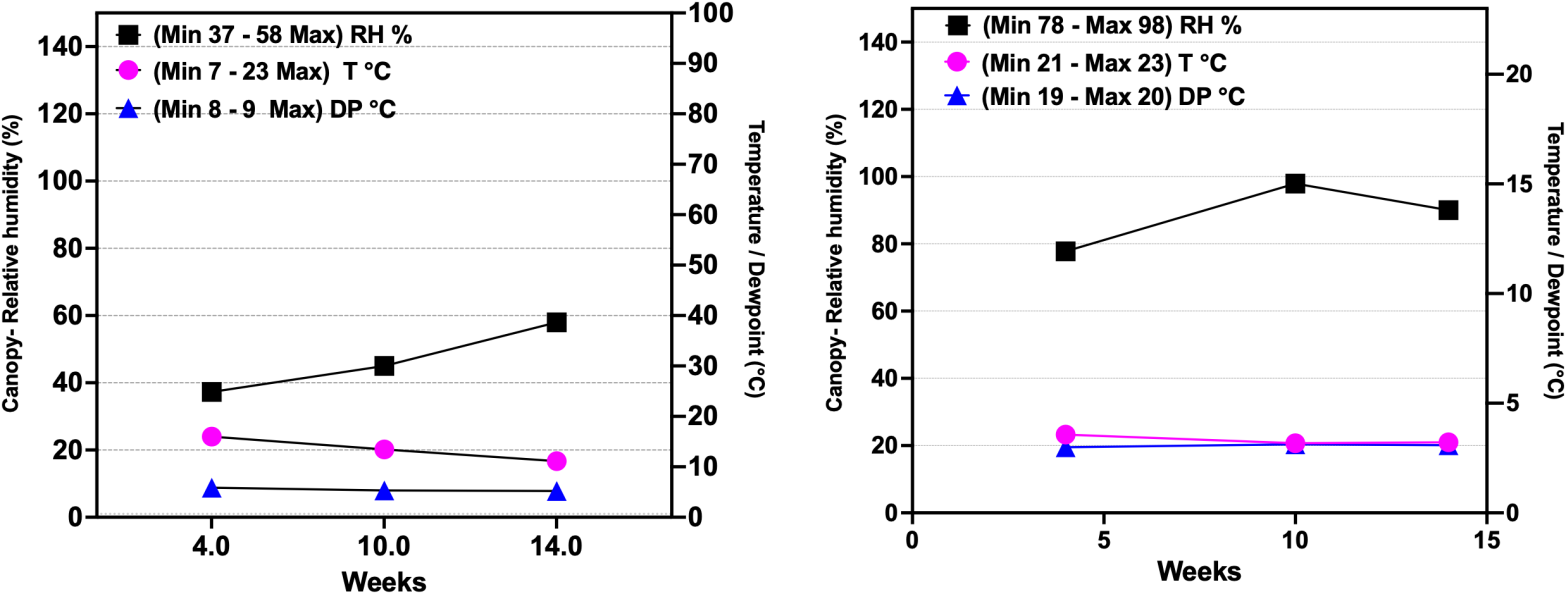
Atmospheric variables measured at the canopy level during the 14th week of the development of the type III (CBD-dominant) cannabis chemotype under controlled conditions, including relative humidity, dew point, and temperature. Labels show the maximum and minimum values recorded in the study. CRH, canopy relative humidity; low RH, 37-58%; high RH, 78-98%; T (°C), temperature; DP (°C), dew point. Based on Corredor Perilla (2024), updated and modified by the author.

In addition to maintaining canopy RH at high (78–98%) and low (37–58%) levels, other atmospheric variables, including dew point (DP) and air temperature (T), were monitored hourly during the 14th week of the genotype’s growth under controlled conditions. Air temperature (T) averaged 20.30 ± 3.60 °C in the low RH range and 21.60 ± 1.40 °C in the high RH range, remaining stable across both humidity conditions (n = 105 days, *p* > 0.005). The maximum and minimum values for T were 7 - 23 °C for the low RH range and 21-23 °C for the high RH (**Figure 1**). The DP averaged 8.20 ± 0.50 °C in the low canopy RH and 20 ± 0.40 °C in the high RH (n = 105 days, *p* < 0.0001). The maximum and minimum values for DP were 8 - 9 °C for the low RH range and 19-20 °C for the high RH (**Figure 1**). No significant differences were observed in soil conductivity or soil temperature between the two RH ranges (n = 56, *p* > 0.0001) (**Supplementary Table 2**).

High RH (78-98%) resulted in low VPD values of 0.05 kPa and 0.25 kPa (Equations 1, 2) during the later vegetative (8 weeks of duration) and flowering stages (6 weeks of duration) of the genotype. Similarly, the leaf VPD (LVPD) was recorded at 0.13 kPa and -0.04 kPa under high RH conditions (**Table 1**). The dew points also increased, reaching 20.40°C and 20.10°C during the vegetative and flowering stages (Equation 3) (**Table 1**). The low RH (37-58%) yielded VPD values of 1.29 kPa and 0.92 kPa, with a minimum LVPD of 1.02 kPa and 0.60 kPa during the vegetative and flowering stages of the genotype (**Table 1**).

**Table 1:** Vapor pressure deficit, leaf vapor pressure deficit, and dew point in the type III (CBD-dominant) chemotype in low RH at the canopy (37-57%) and high RH (78-98%) at the canopy level after 14th weeks of plant growth in controlled conditions (Equations 1–3).

Low RH 37-58%	CRH (%)	Temperature (°C)	Dew point (°C)	VPD kPa	LVPD kPa
Initial Vegetative Stage	37.3	24	8.8	1.87	1.04
Later Vegetative Stage	45	20.2	8	1.29	1.02
Flowering Stage	58	17	7.8	0.92	0.6

High RH 78-98%	CRH (%)	Temperature (°C)	Dew point (°C)	VPD kPa	LVPD kPa
Initial Vegetative Stage	77.8	23	19.5	0.62	0.41
Later Vegetative Stage	97.9	20	20.4	0.05	0.13
Flowering Stage	90	21	20.1	0.25	-0.04

CRH, canopy relative humidity, VPD, vapor pressure deficit, LVPD, leaf vapor pressure deficit, (°C), degrees Celsius, kPa, kilopascals. Based on Corredor Perilla (2024), updated and modified by the author.

### 3.2 High relative humidity impacts the genotype biomass and morphometric traits

**Figure 2:**
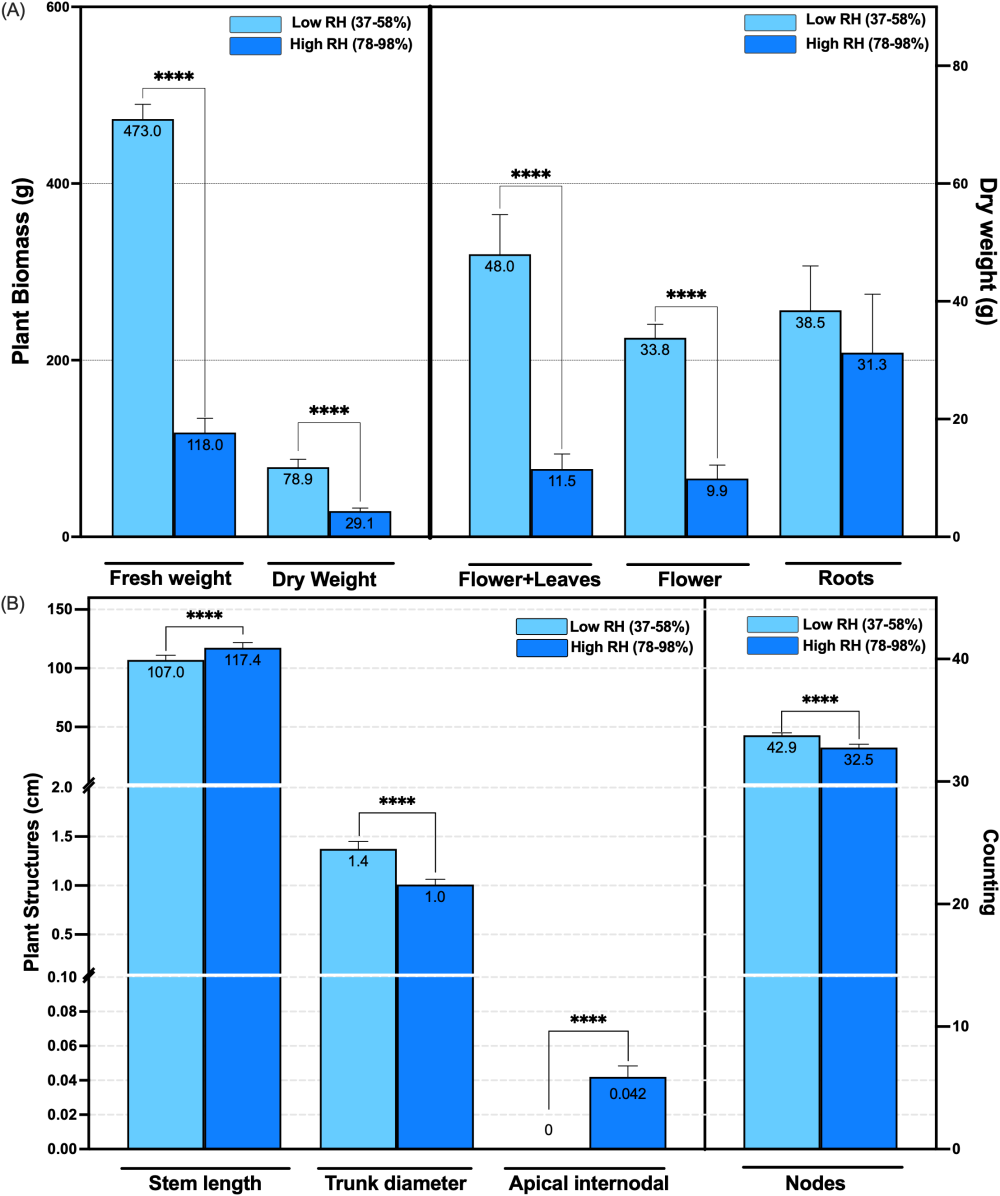
Effect of canopy relative humidity ranges on the type III CBD-dominant chemotype structures after 14 and 15 weeks of plant growth in controlled conditions. **(A)** Fresh and dry biomass weight (g per plant) at low (37-58%) and high (78-98%) canopy relative humidity. This includes biomass of plant structures such as flowers & leaves, flowers, and roots. **(B)** Measurements of plant structures: stem length, trunk diameter, and apical internodal length (cm), as well as the number of nodes in both humidity ranges. **** *p* < 0.0001. Based on Corredor Perilla (2024), updated and modified by the author.

The dry biomass of the genotype, including floral, stem, and foliar tissues, was significantly influenced by RH conditions. Under low RH (37-58%), the average dry weight of flowers plus leaves was 48 g ± 6.70, and flowers averaged 33.80 g ± 2.20. In contrast, plants grown under high RH (78-98%) produced significantly lower flower plus leaves, and flower biomass, measuring 11.50 g ± 2.60, and 9.90 g ± 2.20, respectively (n = 10, *p <* 0.0001) (**Figure 2A**). This represents a 76% increase in floral plus leaves biomass and a 71% increase in flower biomass under low RH compared to high RH. Overall, total dry biomass was 2.71 times greater under low RH conditions (n = 10, *p <* 0.0001) (**Figure 2A**). Root biomass did not significantly change between the low and high RH (n = 10, *p >* 0.0001) (**Figure 2A**).

**Figure 3:**
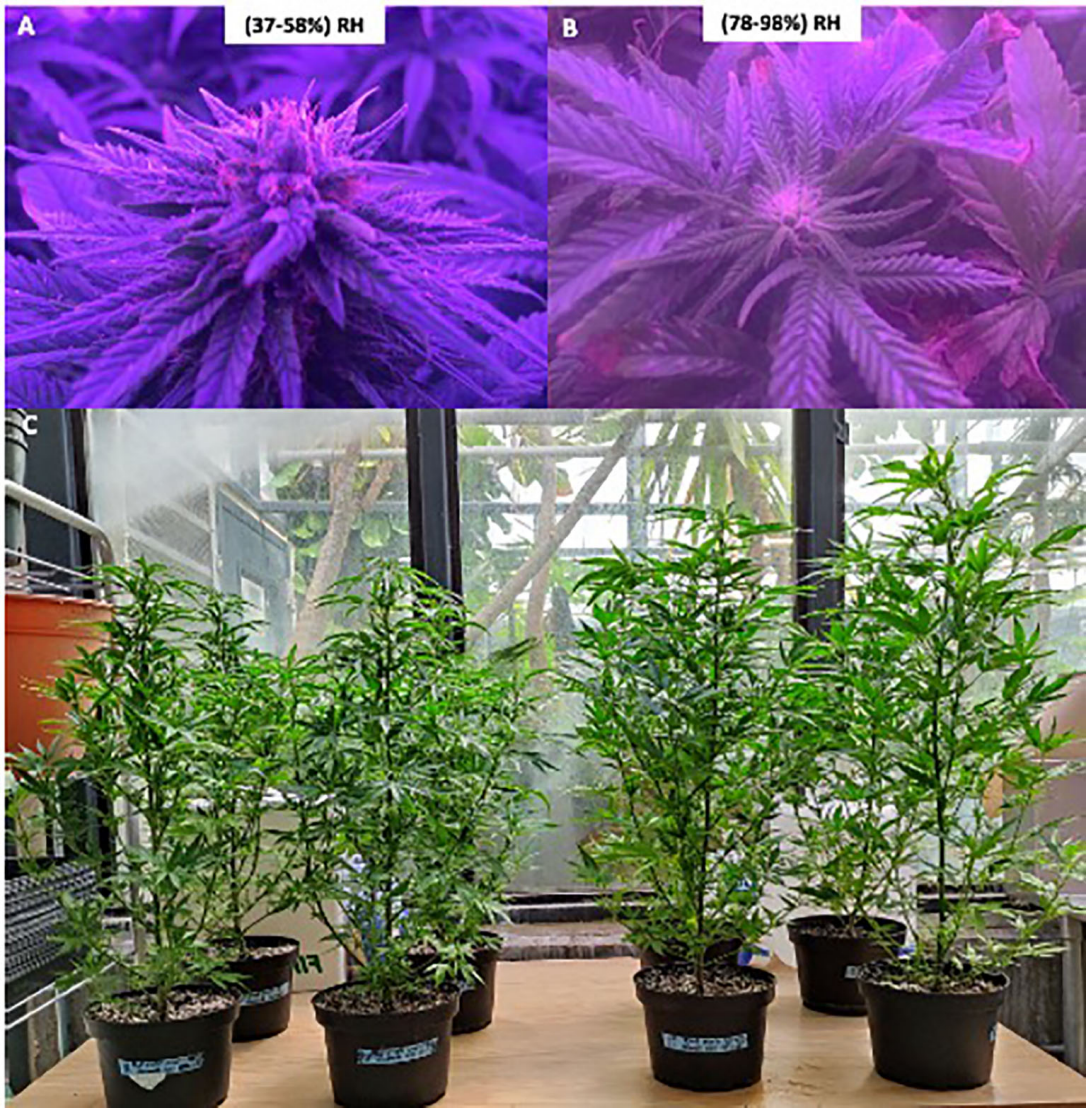
Development of Type III CBD-dominant chemotype during the 14th and 15th weeks in low RH (37-58%) and high RH (78-98%) at the canopy, in controlled conditions. **(A, B)** The flowering stage in both humidity conditions exhibits different inflorescence development at low humidity (37-58%) and high RH (78-98%). **(C)** Plants showing differences in plant height between low RH (37-58%) and high RH (78-98%). RH: Relative humidity. Based on Corredor Perilla (2024), updated and modified by the author.

Morphological traits also varied substantially in response to humidity treatment. The genotype plants grown under low RH exhibited a greater stem diameter (1.40 cm ± 0.07) and a higher number of nodes (43 ± 2.10) than those under high RH, which averaged 1.00 cm ± 0.05 in stem diameter and 32 ± 2.70 nodes (n = 10, *p* < 0.0001) (**Figures 2B**, **3C**, **Supplementary Figure 1**). Conversely, elongation of the apical region was more pronounced under high RH, with the upper three-fourths of the main stem averaging 117 cm ± 4.30, compared to 107 cm ± 4.00 in low RH (n = 10, *p* < 0.0001) (**Figures 2B**, **3A, B**, **Supplementary Figure 1**).

### 3.3 High relative humidity at the canopy level delayed the flowering time

**Figure 4:**
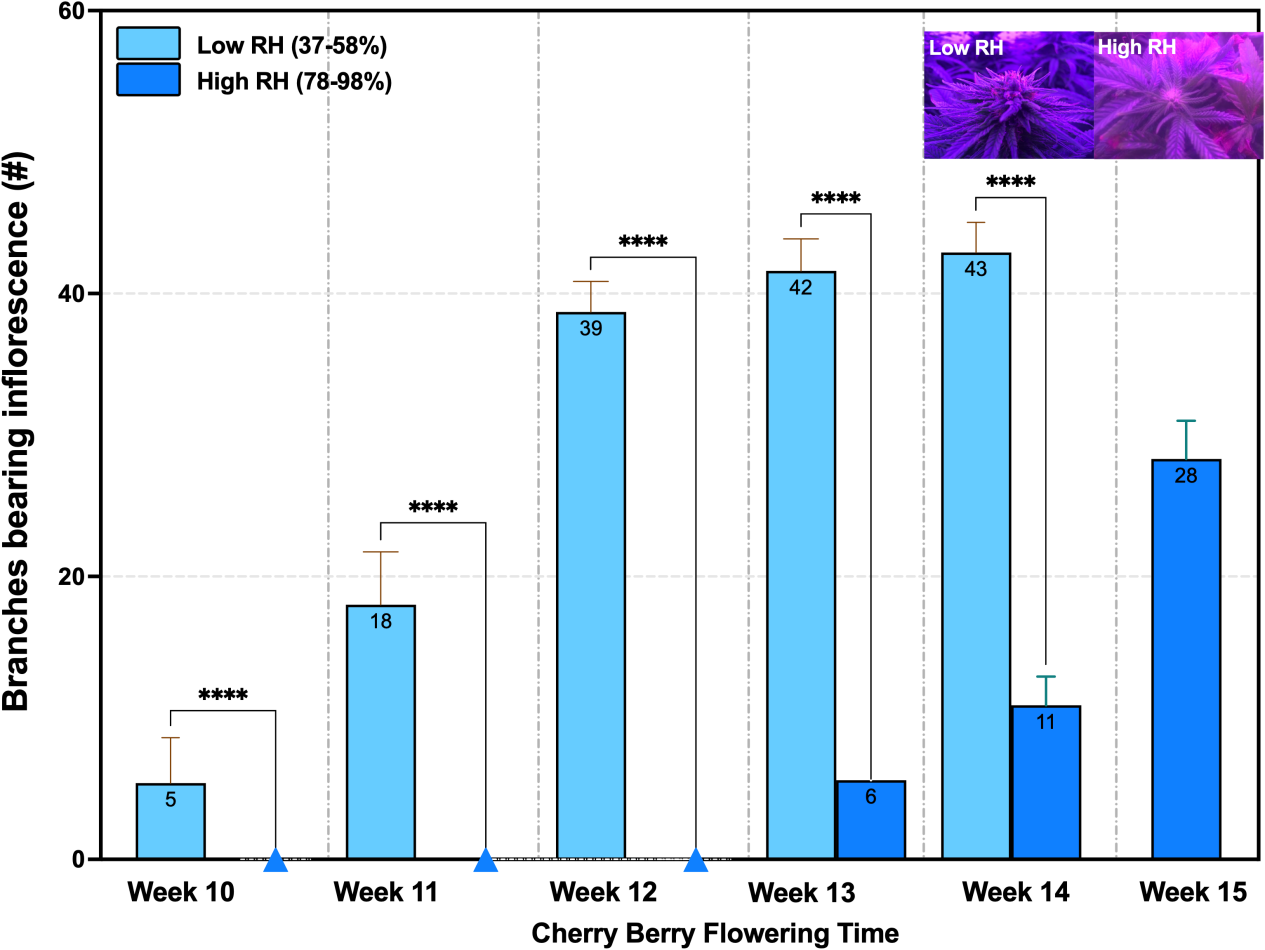
Cannabis flowering development under low RH (37-58%) and high RH (78-98%) showed significant differences in the number of branches with inflorescence development (Mann-Whitney Test, n = 10, ****p < 0.0001) from the 10th to the 14th and 15th weeks. RH: Relative humidity.

Under controlled environmental conditions, the genotype exhibited distinct differences in observable floral development depending on RH. At a low RH, flowering was observable in week 10 with visible shoot apex differentiation. By week 11, bract emergence and pistil development were evident. Inflorescence expansion continued through weeks 12 and 13, during which approximately 40% glandular trichomes exhibited oxidation. By week 14, when plants reached condensed floral maturity and 60–70% of the inflorescence displayed amber coloration, they were harvested (**Figure 4**).

In contrast, plants grown under high RH showed delayed phenological progression. Flowering onset was not observed until week 13. followed by bract and pistil development in week 14. By week 15, only 20–30% trichome oxidation has occurred. Harvest was delayed until week 15 to ensure sufficient floral biomass for cannabinoid quantification and to mitigate guttation-related tissue damage, which spread more aggressively under high humidity conditions (**Figure 4**).

Notably, inflorescence formation per branch was significantly reduced under high RH compared to low RH. Quantitative analysis revealed decreases of 14.30% and 25.60% in weeks 13 and 14, respectively, indicating a marked suppression of floral development in elevated humidity environments (n = 10, *p* < 0.0001) (**Figure 4**).

### 3.4 Cannabinoid concentrations drastically decreased in high relative humidity

**Figure 5:**
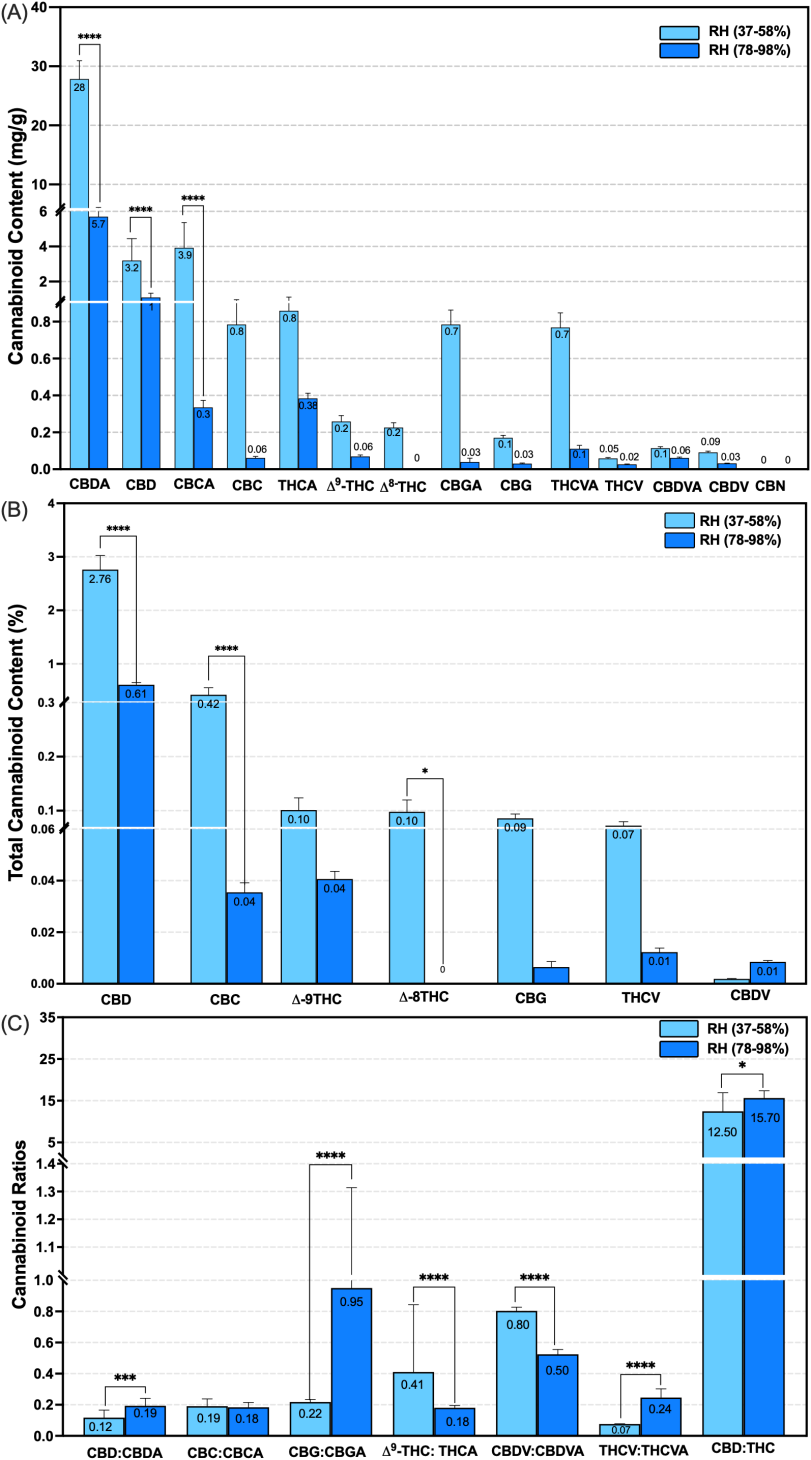
Effect of canopy relative humidity ranges on total cannabinoid concentration, after decarboxylation, and cannabinoid ratios in the type III CBD-dominant strain following 14 (low RH) and 15 (high RH) weeks of plant growth under controlled conditions. Cannabinoids were assessed in low and high canopy RH ranges (n = 10, *p <* 0.0001). **(A)** Comparisons of cannabinoid concentration before decarboxylation in mg g^-1^ per plant at the evaluated humidity ranges; **(B)** Comparison of total cannabinoid percentage after decarboxylation at the assessed humidity ranges (Equation 4). **(C)** Comparison of cannabinoid ratios at the evaluated humidity ranges. RH, Relative Humidity; Acidic and Neutral cannabinoids: Cannabidiol (CBD-A/CBD), Cannabichromene (CBC-A/CBC), Tetrahydrocannabinol (THC-A/Δ^9^-THC), isomers of THC (Δ^8^-THC), Cannabigerol (CBG-A/CBG), THCV, Tetrahydrocannabivarin, CBDV, Cannabidivarin, Oxidized and degraded molecules: Cannabichromene (CBN). **** *p <* 0.0001, * *p < 0.01.* Based on Corredor Perilla (2024), updated and modified by the author.

To assess the impact of canopy-level RH on cannabinoid composition, a total of 14 phytocannabinoids were quantified under low (37-58%) and high (78-98%) RH conditions during the flowering stage. Cannabinoids predominantly remained in their acidic forms by weeks 14 and 15 across both humidity ranges (Equation 4) (**Figures 5A, C**).

Exposure to high RH resulted in substantial reductions in key acidic and neutral cannabinoids. Compared to low RH content, the CBD-A decreased by 20.50%, from 27.80 mg g^-1^ to 5.70 mg g^-1^, CBD exhibited a 31.20% reduction, from 3.20 mg g^-1^ to 1.0 mg g^-1^, and CBC-A declined by 7.70%, from 3.90 mg g^-1^ to 0.30 mg g^-1^ (n = 10, *p* < 0.0001) (**Figure 5A**). Following decarboxylation, total neutral cannabinoid concentration also showed a significant reduction under high RH. CBD content declined from 2.80% ± 0.20 to 0.61% ± 0.03, and CBC decreased from 0.42% ± 0.13 to 0.04% ± 0.003 (n = 10, *p* < 0.0001) (Equation 4) (**Figure 5B**).

The ratio of acidic to neutral forms also varied markedly between the RH treatments. Under low and high RH, the ratios were elevated for CBD from 0.12 ± 0.04 to 0.19 ± 0.04, CBG from 0.22 ± 0.01 to 0.95 ± 0.30, THCV from 0.07 ± 0.001 to 0.24 ± 0.05, respectively. In contrast, other cannabinoid ratios decreased, such as Δ^9^-THC from 0.41 ± 0.40 to 0.18 ± 0.01, and CBDV from 0.80 ± 0.02 to 0.50 ± 0.03 (n = 10, *p* < 0.0001) (**Figure 5C**). No significant changes were observed when comparing CBC ratios across humidity conditions. Notably, the oxidative cannabinoid Δ^8^-THC was undetected under low RH conditions, and no degradation products such as CBN were observed under either humidity regime (**Figures 5A, C**).

## 4 Discussion

Relative humidity is a key environmental factor that influences the growth, productivity, and secondary metabolism of *C. sativa*, including cannabinoid biosynthesis. While general cultivation guidelines recommend maintaining RH within the range of 55% to 60% during vegetative and flowering stages (Das et al., 2022; Jin et al., 2019), the effects of high canopy-level RH on genotype-specific morphology and cannabinoid concentration remain largely unexplored. Understanding these responses is essential for optimizing production under variable humidity conditions.

One crucial factor influenced by RH is the vapor pressure deficit (VPD), which governs the driving force for transpiration and plays a key role in plant water relations and metabolic efficiency. For *C. sativa*, optimal VPD ranges are generally reported as 0.50–1 kPa during cloning and seedling stages, 0.70 -1.20 kPa during the vegetative stage, and 1.00 -1.50 kPa during flowering (Breit et al., 2019; Galindo et al., 2023; Vernon et al., 2023).

In the present study, cannabis plants grown under high RH exhibited markedly reduced VPD values, remaining below optimal thresholds by 89% during the vegetative stage and 75% during flowering. Sustained low VPD can limit transpiration, impair nutrient uptake, and reduce photosynthesis efficiency, while also increasing vulnerability to pests and diseases (Ding et al., 2022; López et al., 2021). These findings underscore the importance of understanding how high-humidity environments alter VPD dynamics and their broader impact on cannabis physiology and productivity.

The impact of high canopy RH on the flowering stage of the genotype was particularly evident. Under low RH conditions, flowering nutrition and photoperiod treatments began in week nine, with visible floral initiation, specifically shoot apex differentiation, occurring by week 10. In contrast, high RH conditions delayed the onset of flowering until week 13. This delay suggests that reduced transpiration associated with low VPD may impair the transport of essential nutrients, such as phosphorus and magnesium, which are critical for flower development (Chia and Lim, 2022; Ding et al., 2022). Additionally, increased stomatal aperture under high RH can disrupt internal water flow and nutrient homeostasis, further inhibiting reproductive progression. Similar effects have been observed in *Chrysanthemum morifolium*, where low VPD delayed flowering by approximately four days (Mortensen, 1986, 2000).

The effects of high RH extended beyond flowering delays to changes in plant morphology. Compared to low RH, this genotype exhibited a significant increase in apical internode length (from 0 mm to 0.42 mm) and stem length (from 107 cm to 117.4 cm). Similar responses have been reported in ornamental crops, such as chrysanthemum, kalanchoe, and poinsettia, where increased humidity promotes stem elongation (Mortensen, 2000). This is likely due to the search for radiation in high-humidity environments to increase photosynthesis and nutrient allocation, contributing to elongated apical and stem growth. The dynamic interplay between nutrient allocation and hydraulic functioning under elevated humidity conditions has also been documented in silver birch trees (Sellin et al., 2015), suggesting a broader physiological relevance to these findings. At the canopy level, the presented results demonstrate that elevated RH ranging from78% to 98% promotes stem elongation and increased internodal spacing in the studied genotype. Nevertheless, optimal plant density remains a critical determinant for achieving substantial foliar biomass accumulation.

Furthermore, elevated RH significantly influenced aerial shoot structures and perturbed the soil-water balance, resulting in anoxic conditions within the root zone. Such hypoxic stress is known to induce hormonal signaling cascades involving ethylene and gibberellins (GA), which facilitate physiological adaptations to low-oxygen environments (Waadt et al., 2022). In flood-adapted rice, GA promotes stem elongation by stimulating internodal growth, enabling the plant to maintain access to atmospheric oxygen during submergence (Panda and Barik, 2021). It is plausible that analogous mechanisms are at play in this genotype, where atmospheric water saturation may activate ethylene signaling pathways and GA biosynthesis, thereby facilitating stem elongation and internode expansion under high humidity stress.

Although elevated RH stimulated stem elongation in this genotype, it had a detrimental effect on biomass accumulation. Both fresh and dry biomass were significantly reduced across all plant organs under high RH conditions (78-98%). Comparable reductions in biomass have been reported in CBG-dominant hemp varieties cultivated in Florida’s high-humidity environment, underscoring the critical influence of atmospheric moisture on overall plant productivity (Chiluwal et al., 2023). These observations are consistent with findings in other species, such as Begonia, where excessive RH similarly limited biomass accumulation (Mortensen, 2000). However, not all stressors exert equivalent effects; for example, drought stress in *C. sativa* chemotype II did not alter the dry weight of inflorescences (Caplan et al., 2019), highlighting the distinctive physiological consequences of high humidity stress on biomass production.

High RH also influenced key structural traits, including trunk diameter. In the studied genotype elevated RH conditions were associated with a reduction in trunk thickness (**Figure 2B**), paralleling findings in *Betula pendula* Roth, where stem diameter decreased due to reduced mechanical loading in high-humidity environments (Sellin et al., 2015). This observation supports the hypothesis that during the vegetative stage, high RH may increase root hydraulic conductance in this cannabis genotype, contributing to altered stem development and reduced root growth.

Another notable response to high RH was a reduction in the number of nodes and lateral branches in the studied genotype. This decline is likely attributable to guttation observed in lower canopy, which led to premature leaf senescence during the flowering stage (weeks 10 to 14) (**Figure 4**). The phenomenon aligns with findings in silver birch trees, where increased root hydraulic conductance under humid conditions was associated with a decrease in leaf area, promoting stem elongation and extended internodal length (Sellin et al., 2015). Supporting this, studies on guttation in various crops under low VPDs and high RH have shown that the accumulation and breakdown of guttation exudates (e.g., minerals, hormones, enzymes, etc.) can discolor leaf tips from green to yellow, followed by necrosis and senescence, particularly in older foliage (Singh, 2014; Zheng et al., 2021).

Beyond its adverse effects on biomass and morphology, high RH had a pronounced impact on cannabinoid concentration in this cannabis genotype. Elevated humidity delayed the onset of flowering by approximately three weeks and significantly reduced the concentration of total CBD and CBC, as well as their acidic precursors, CBD-A, CBC-A (**Figures 5A, B**). These findings are consistent with previous studies, which have shown that environmental stressors reduce cannabinoid yield. For instance, under drought conditions, the early flowering stage of the CBD genotype ‘Green-Thunder’ exhibited reduced THC and CBD levels (Park et al., 2022). Similarly, a six-year field study involving eight industrial hemp varieties reported diminished CBD content following periods of precipitation, although no significant cannabinoid changes were detected within a moderate RH range of 45–65% (Sikora et al., 2011). Likewise, broader climatic factors such as wind and flood have been shown to reduce phytochemical diversity and shift cannabinoid concentration, primarily leading to reductions in CBD content (Kay et al., 2025). Collectively, these findings suggest that abiotic stressors, including drought, excessive rainfall, wind, flooding, and high relative humidity, can impair cannabinoid biosynthesis in a genotype-specific manner, with outcomes strongly influenced by environmental conditions, cultivation practices, and the timing of stress relative to anthesis.

Elevated RH also influenced cannabinoid composition, particularly the ratios between acidic and neutral forms (e.g., CBD: CBD-A and CBG: CBG-A), as well as the CBD: THC ratio, all of which were higher under high RH conditions compared to low RH (**Figure 5C**). This increase may be attributed to a delayed flowering onset—observed as a three-week shift post-anthesis—followed by the inflorescence harvest. The extended flowering duration likely allowed for additional decarboxylation, contributing to elevated ratios of neutral cannabinoids.

In support of this, several field-based studies conducted in high-humidity regions or seasons have reported that cannabinoid concentration during flowering vary depending on both genotype genetics and environmental conditions. These studies documented shifts in the CBD: THC ratio in outdoor-grown plants, further highlighting the interaction between genotype response and environmental context (Chiluwal et al., 2023; Stack et al., 2021; Trancoso et al., 2022; Yang et al., 2020). Such variability underscores the importance of standardizing cannabinoid profiling across developmental stages under defined environmental and cultivation parameters to optimize yields and accurately identify peak cannabinoid concentration windows.

Notably, this study also revealed a substantial decline in CBC levels under high RH, with levels dropping from 0.42% in low RH to 0.04% in high RH (**Figure 5B**). Existing predictive and real-time studies suggest that CBC accumulation is favored by an extended vegetative phase and an earlier harvest during flowering (Naim-Feil et al., 2023).The impact of high RH significantly altered the VPD values, influencing growth, morphology, and cannabinoid composition of this genotype. In this study, the vegetative stage lasted eight weeks, which is slightly longer than typical growth chamber conditions, and may have affected the cannabinoid composition. Further research is needed to determine optimal harvest timing and environmental conditions for maximizing specific cannabinoids under different humidity regimes.

Because this study focused on a single genotype, future research should investigate the effects of elevated humidity across multiple CBD-rich hemp varieties. This work will help identify differences in growth, morphology, cannabinoid concentration, and physiology, providing critical insights for selecting climate-adaptive genotypes and developing region-specific management strategies to improve cannabis production amid increasing climate variability.

## 5 Conclusions

This study is the first to examine how two different canopy-level relative humidity (RH) ranges impact morphology, biomass, and cannabinoid concentration in CBD-rich hemp grown under controlled conditions. Elevated RH significantly altered plant structure, reduced biomass, and hampered inflorescence development, while low vapor pressure deficit (VPD) caused physiological stress symptoms such as guttation, tip burn, and leaf rot. These combined effects delayed flowering and lowered overall cannabinoid concentration, particularly CBD and CBC.

The low RH range used in this study reflects typical humidity levels during cannabis cultivation seasons in Colorado and was chosen as the control condition. However, this range should not be seen as the ideal environment for cannabis growth. While it offers a realistic baseline for comparison, additional research is necessary to see if slightly higher or lower RH levels could improve plant health and maximize cannabinoid production across different genotypes.

Relative humidity is a crucial factor affecting VPD and must be carefully managed throughout cannabis growth. If environmental conditions are not consistent at each stage, VPD imbalances can harm plant physiology, morphology, and cannabinoid concentration. Further research is needed to identify optimal RH ranges and temperatures that maximize cannabinoid concentrations across different genotypes.

Since cannabis is cultivated in various production systems, understanding humidity thresholds is essential for selecting climate-adapted genotypes and developing effective management strategies. Automated humidity control and strategic plant spacing can improve airflow, reduce disease risk, and boost yields, leading to more sustainable and resilient cannabis cultivation.

## Data Availability

The datasets presented in this study can be found in online repositories. The names of the repository/repositories and accession number(s) can be found below: This manuscript is an updated and revised version of the original submitted as a requirement for Corredor-Perilla, Ingrid Carolina’s doctorate in Agroecology. It will be fully published on October 18, 2026, in the digital library of the National University of Colombia repository at https://repositorio.unal.edu.co/handle/unal/87226, under the Creative Commons (Attribution–Noncommercial–Non-Derivatives 4.0 International (CC BY-NC-ND 4.0) license )” license. The updated information can be found in the article and the **Supplementary Material**. Further questions can be directed to the corresponding author.
