# Supplementary material for: Elevated relative humidity significantly decreases cannabinoid concentrations while delaying flowering development in Cannabis sativa L
Source: Front Plant Sci. 2025 Nov 17;16:1678142. doi: 10.3389/fpls.2025.1678142 (PMC12666426; doi:10.3389/fpls.2025.1678142)
Supplement: Supplementary file 1 [file DataSheet1.docx]

Supplementary Material

**Supplementary Table 1.** Nutritional Program of cv. Cherry Berry during 14 and 15 weeks. Abbreviations: NPK: nitrogen, potassium, and phosphorus. Based on Corredor Perilla (2024), updated and modified by the author.

| **Nutritional Regimen** | **Doses Applied** | **Frequency of Application** | **Volume**  **(mL)** | **Compounds** |
| --- | --- | --- | --- | --- |
| **Aeroponics**  **Root formation** | Clonex Rooting Gel, 100 ml | 2 times a week  If no root formation appeared | Dipping the bottom meristem in 4 ml of Clonex rooting gel | Indole-3-Butiric Acid: 0.31%, Other ingredients 99.9% |
|  | Foliar Nutrition  Hydrodynamics Clonex Mist^®^, 750 mL | 2 times a week  For 4weeks  (including acclimation in soil substrates. For 2-weeks) | Spray flush covering the canopy area of the cuttings | Total nitrogen: 1%, 0.05% ammoniacal nitrogen, 0.95% nitrate nitrogen, available phosphate(P_2_O_5_), soluble potash (K_2_O). |
|  | **Early vegetative growth:**  1.25 ml/15L  **Late vegetative growth:**  1.25 ml/15L  **Bloom growth:**  1.25 ml/15L | 2 times a week  For last 2 weeks of root formation, and 2 weeks acclimation | The nutritional solutions were added to only one nutritional regimen in reverse osmotic water.  pH oscillation was between 5.5 and 6.5 | Nutritional Package Regimen:  -Flora Gro Doses NPK (2-1-6):  -Flora Micro NPK (5-0-2) 1  -Flora Bloom NPK (0-5-1) |
| **Commercial soil substrates**  **for soil pots** | Pro Mix Hp ^®^  151.41/  45 kg/ L | Once during the 14th weeks of plant growth in 20 soil pots of  3.78 ml | Mixed soil substrate with a  pH 5.5-7.0  Osmotic Reverse Water pH 7.0 | Sphagnum Peat moss/ ground stone dolomite/ coir base- Mycorrhizae |
|  | Fox Farm Ocean Forest ^®^  18.14 kg |  |  | Earthworm castings and Mycorrhizal fungi |
|  | Roots Organic Original ^®^ 10.8 kg |  |  | Organic Material and Mycorrhizal fungi |
|  | Wiggle Worm Soil Builder^®^ 30 13.61Kg |  |  | Pure worm soil builder |
|  | Botanicare CocoGro Loose^®^  28 kg/l |  |  | Coir fiber |
| **Soluble Potash**  **ArmorSi^®^** | **Early vegetative growth:**  10 ml/15L  **Late vegetative growth:**  20 ml/15L  **Bloom growth:**  20 ml/15L  Water flush  (last week of flowering) | 2 times a week | The nutritional solutions were added to only one nutritional regimen in reverse osmotic water.  Each Plant was fed with 900 ml per plant.  pH oscillation was between 5.5 and 6.5 | Silicon dioxide 10% derived from potassium silica |
| **Calcium & Magnesium**  **CaliMagic^®^** | **Early vegetative growth**:  10 ml / 15L  **Late vegetative growth:**  20 ml/ 15L  **Bloom growth:**  30 ml/15L  Water flush  (last week of flowering) |  |  | Calcium and Magnesium nitrate (Water soluble magnesium), Iron DTPA. Total Nitrogen (1%), Calcium (5%), Magnesium (1.5%), Chelated Iron (0.1%) |
| **FloralGro^®^ NPK (2-1-6)** | **Early vegetative growth:** 11 ml/ 15L  **Late vegetative growth:**  20 ml/ 15L  **Bloom Growth:**  10ml/15L  Water flush  (last week of flowering) |  |  | Total Nitrogen:2%  (Ammoniacal Nitrogen 0.025%, Nitrate Nitrogen:1.75%:) available Phosphorus (P_2_ O_5_): 1%, soluble Potash (K_2_O):6%, Magnesium: 0.5% water soluble Magnesium |
| **FloralMicro^®^**  **NPK (5-0-1)** | **Early vegetative**  **growth**: 10 ml/ 15L  **Late vegetative Growth**: 20ml / 15L  **Bloom Growth**  10mL/15L  Water flush  (last week of flowering) |  |  | Total Nitrogen: 5%, Soluble Potash: 1%, Calcium :5%, Boron:0.1%, Cobalt: 0.0005%, Copper: 0.01% chelated copper, Iron chelated 0.1%, Molybdenum 0.0008%, Chelated manganese 0.05%, chelated zinc 0.015% |
| **FloralBloom^®^**  **NPK (0-5-4)** | **Early vegetative Stage:**  10 ml/15L  **Late vegetative**  **growth**:  20 ml/ 15L  **Bloom Growth**  40 ml/15L  Water flush  (last week of flowering) |  |  | Available Phosphorus: 5%, Soluble potash: 4%, Magnesium: 1.5% (water soluble magnesium), Sulfurs:1% (combined sulfur) |
| **Compost Tea**  **Recharge^®^** | Early Vegetative Stage in soil pots  5ml/3.78L | Once a week for 3 weeks | Dissolved in reverse osmotic water. pH between 5.5-6.5 | 453 g of Mycorrhizae: *Glomus* spp. 6.4 UFC/g), *Trichoderma*, beneficial bacterium in Kelp (*Bacillus* spp. 1x10 ^9^ cells/g) Humic acids derived from Leonardite 5%, Fulvic acids 3% and Molasses6% |

**Supplementary Table 2.** Soil conductivity and soil temperature in ‘Cherry Berry’ during the 14th weeks of plant development in low RH (37-58%) and high RH (78-98%) at the canopy level (n = 10, *P* > 0.0001). Abbreviations: ns: no significant differences, SD: standard deviation, RH: relative humidity, mS cm^-1^: MiliSiemens per centimeter. Based on Corredor Perilla (2024), updated and modified by the author.

| Below plant measurements at 14 W | Low RH (37-58%) | | High RH (78-98%) | | *P >* 0.0001 |
| --- | --- | --- | --- | --- | --- |
| Soil conductivity  (mS/cm) | Mean | SD | Mean | SD |  |
| Early Vegetative Stage | 0.49 | 0.23 | 0.60 | 0.20 | ns |
| Later Vegetative Stage | 0.86 | 0.45 | 1.02 | 0.28 |  |
| Flowering Stage | 1.32 | 0.97 | 1.65 | 0.24 |  |
| Soil temperature  (℃) |  | | | | |
| Early Vegetative Stage | 19.79 | 0.51 | 20.79 | 1.03 | ns |
| Later Vegetative stage | 18.83 | 1.81 | 18.67 | 1.98 |  |
| Flowering Stage | 18.31 | 2.31 | 18.27 | 1.71 |  |


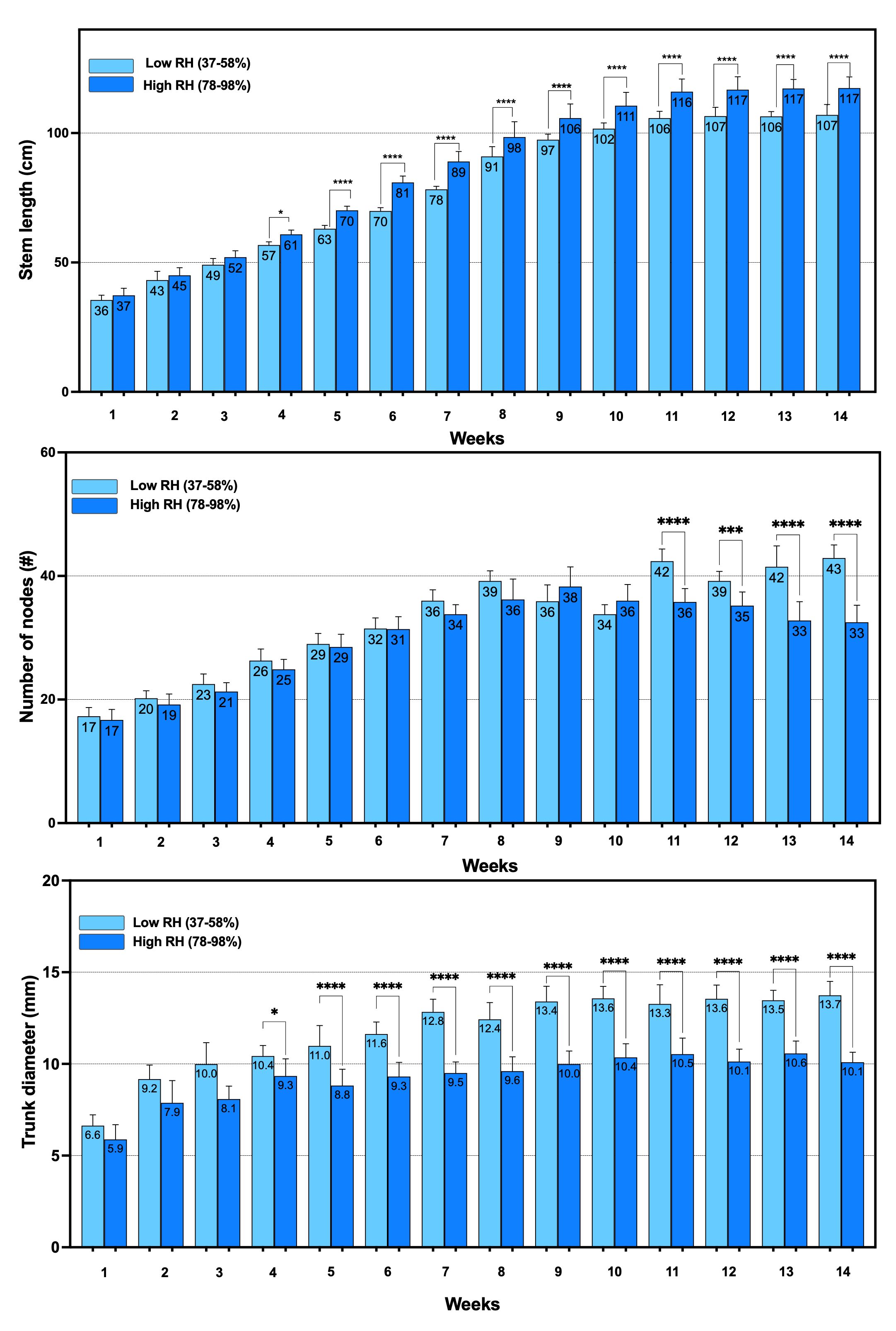


**Supplementary Figure 1.** Morphometric development of ‘CBD-dominant strain’ at the canopy level over the 14th week under low RH (37-58%) and high RH (78-98%). Measurements include stem length, number of nodes, and trunk diameter—abbreviation: RH — Relative humidity. Based on Corredor Perilla (2024), updated and modified by the author.
